# Intramedullary leg lengthening with a motorized nail: Indications, challenges, and outcome in 32 patients

**DOI:** 10.3109/17453674.2011.584209

**Published:** 2011-07-08

**Authors:** Andreas H Krieg, Ulrich Lenze, Bernhard M Speth, Carol C Hasler

**Affiliations:** University Children's Hospital, Basel (UKBB), Switzerland

## Abstract

**Background and purpose:**

In the last decade, intramedullary limb lengthening has become a viable alternative to traditional external systems. We retrospectively analyzed the use of an intramedullary motorized nail (Fitbone) in a consecutive series of 32 patients.

**Patients and methods:**

During the period September 2006 to December 2008, 32 consecutive patients with a median age of 17 (IQR: 15–19) years were treated with a fully implantable, motorized intramedullary lengthening device (Fitbone). The median leg length discrepancy was 35 (IQR: 30–44) mm at the femur (n = 21) and 28 (IQR: 25–30) mm at the tibia (n = 11).

**Results:**

Leg lengthening was successful in 30 of 32 cases, with no residual relevant discrepancy (± 5 mm). No intraoperative complications were observed. The consolidation index was significantly different (p = 0.04) between femoral lengthening (mean 35 days/cm) and tibial lengthening (mean 48 days/cm) but did not depend on age older/younger than 16 or previous operations at the affected site. 3 problems, 3 obstacles, and 4 complications (3 minor, 1 major) were encountered in 8 patients, 5 of which were implant-associated.

**Interpretation:**

This technique even allows correction in patients with multiplanar deformities. Compared to external devices, intramedullary systems provide comfort and reduce complication rates, give improved cosmetic results, and lead to fast rehabilitation since percutaneous, transmuscular fixation is prevented. This results in reasonable overall treatment costs despite the relatively high costs of implants.

Operative leg length equalization includes techniques such as contralateral epiphysiodesis during growth, or shortening and lengthening procedures. In the last decade, lower complication rates and greater patient comfort (Baumgart et al. 1997, Guichet 1999, Cole et al. 2001) have made intramedullary limb lengthening a valuable alternative to traditional external fixation distractor systems (Guichet et al. 2003, Hankemeier et al. 2005, Leidinger et al. 2006, Krieg et al. 2008). Two mechanical devices, namely the Albizzia nail and the ISKD (intramedullary skeletal kinetic distractor) nail, and one motorized nail (Fitbone) have been reported (Betz et al. 1990, Cole et al. 2001, Guichet et al. 2003).

Here we present the indications and limitations, and the results and complications of the Fitbone nail. We identified possible factors that may influence the clinical outcome. In addition, we did a comparative cost analysis of different treatment methods.

## Patients and methods

**Figure 1.**
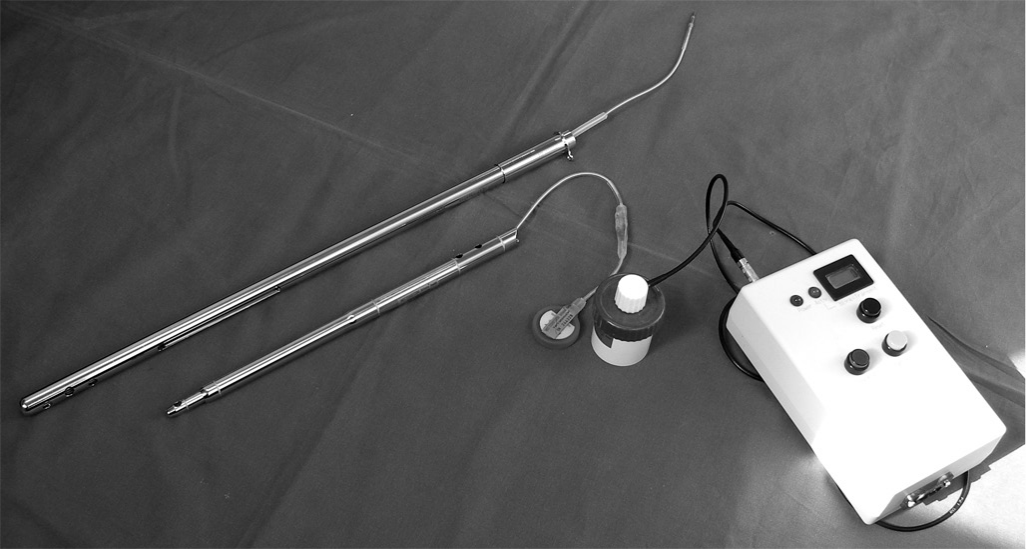
Fitbone TAA (Telescope Active Actuator) and SAA (Sliding Active Actuator) with control and transmitter unit.

During the period September 2006 to December 2008, 32 consecutive patients with a median age of 16.9 (IQR: 15.3–19.4) years were treated with a fully implantable, motorized intramedullary lengthening device (the Fitbone Sliding Active Actuator (SAA) nail and the Telescope Active Actuator (TAA) nail; WITTENSTEIN Intens GmbH, Igersheim, Germany) (Figure 1). The median leg length discrepancy was 35 (IQR: 30–44) mm at the femur and 28 (IQR: 25–30) mm at the tibia.

The underlying pathologies and the number of operations at the affected site are listed in Table 1. None of the cases had a history of osteomyelitis within the previous 2 years, nor showed any reduced bone quality. 9 patients presented with shortening as the only deformity, 14 presented with two deformities, and 9 had three deformities.

**Table 1.** Patient characteristics and results

A	B	C	D	E	F	G	H	I	J	K	L	M	N	O
1	16.0	Femur	Posttraumatic	None	35	35	30	0.85	24	35	8	90		a
2	15.2	Tibia	Congenital	Ext, Vari	30	30	28	0.93	38	50	15		79	
3	19.0	Tibia	Tumor	Flex, Vari	30	27	29	1.07	22	33	14		85	
4	53.0	Femur	Congenital	Ext, Valg	45	45	60	1.33	20	35	14	86		
5	15.9	Femur	Posttraumatic	Ext, Vari	30	25	22	0.88	18	31	14	89	87	
6	15.2	Tibia	Posttraumatic	Valg	30	30	28	0.93	35	48	13		92	b
7	15.0	Tibia	Congenital	Ext	25	25	27	1.08	36	49	14		86	
8	15.7	Femur	Congenital	None	25	27	27	1	17	27	10	86		
9	19.5	Femur	Tumour	Valg	40	40	35	0.87	19	29	10	89		
10	14.0	Femur	Posttraumatic	None	40	38	29	0.76	16	25	10	91		
11	34.5	Femur	Posttraumatic	None	40	38	32	0.84	16	27	12	92		
12	19.1	Femur	Congenital	Ext, IR	30	30	29	0.96	23	35	12	88		c
13	16.5	Femur	Tumor	Ext, IR	27	27	33	1.22	44	51	10	90		
14	19.3	Tibia	Congenital	Valg	28	31	35	1.12	77	133	14		86	d
15	14.4	Tibia	Congenital	Flex	25	26	27	1.04	40	65	7		88	e
16	15.3	Femur	Congenital	None	30	30	27	0.9	36	47	10	86		
17	16.8	Tibia	Congenital	Valg, ER	23	22	22	1	51	63	9		92	
18	17.5	Femur	Congenital	Vari, IR	45	42	48	1.14	16	29	12	89		
19	17.2	Femur	Posttraumatic	None	30	27	28	1.03	36	49	9	88		
20	16.3	Femur	Congenital	Vari	35	37	30	0.81	16	26	9	92		
21	15.3	Femur	Infection	Vari	60	60	87	1.45	25	27	10	86		
22	19.9	Femur	Posttraumatic	None	35	35	29	0.82	30	41	10	92		
23	14.4	Femur	Infection	Valg	80	80	82	1.02	26	27	14	102		
24	17.9	Tibia	Congenital	Vari	43	43	61	1.41	22	37	12		91	f
25	14.3	Femur	Congenital	Vari	55	55	51	0.93	30	41	14	86		
26	33.9	Tibia	Idiopathic	None	25	22	30	1.36	30	48	14		93	
27	11.5	Tibia	Infection	Valg, ER	51	54	49	0.9	13	24	12		86	
28	19.0	Tibia	Neurologic (hemiparesis)	Var	25	25	38	1.52	14	34	14		89	g
29	15.8	Femur	Congenital	Vari	40	40	34	0.85	17	20	11	90		
30	28.1	Femur	Congenital	Valg	25	20	29	1.45	30	54	15	87		h
31	19.1	Femur	Posttraumatic	IR	43	43	44	1.02	24	37	9	86		
32	18,8	Femur	Posttraumatic	None	24	21	35	1.66	37	62	9	88		
Mean 19.3				35.9	35.3	37.3	1.06	27	41.8	11.6	89.2	87.9		
Median 16.8				30	30.5	30	1.01	24	36.1	12.0	89.0	88.0		

A Patient B Age C Site D Diagnosis E Additional deformity correction Ext extension ER external rotation Flex flexion IR internal rotation Valg valgisation Vari varisation F Target length, mm G Length achieved, mm H Distraction period, days I Distraction index, mm/day J Maturation index, days/cm K Consolidation index, days/cm L Hospital stay, days M Postop. mat. dist femur angle (LDFA), degrees N Postop. med. proximal tibia angle (MPTA), degrees O Complications

a Motor failure, device exchange

b Deep vein thrombosis

c Knee extension contracture

d Bolt loosening, running back 10 mm, insufficiant callus formation

e Bolt loosening, running back 5 mm

f Increase of pre-existing equinus deformity, delayed callus formation

g Bolt loosening

h Bolt loosening; running back 7 mm

The indication for surgery was based on a minimum LLD of more than 2 cm, clinical/functional symptoms, and ability to independently handle the lengthening device. Lengthening for cosmetic reasons only was not accepted.

The SAA, which is restricted to femoral use, is mostly implanted through an antegrade approach. As the SAA features a slotted sliding hole in its middle part, this device allows lengthening as well as bone transport. TAA nails are available for both the femur and the tibia. They are implanted through a retrograde approach at the femur and an antegrade approach at the tibia. The femoral TAA is a straight nail; the tibial one is available as either a straight nail or one with a proximal Herzog angulation. In contrast to the SAA, lengthening with the TAA is accomplished by a telescoping mechanism. Various sizes, different designs, and even custom-made implants of both nail types are available.

Preoperatively, standardized anteroposterior and lateral long standing radiographs of both legs were taken and a preoperative planning was performed, based on the protocol for retrograde corrective planning described by Baumgart (2009). Mechanical axis alignment, LLD and projected joint angles, such as the mechanical lateral distal femoral angle (mLDFA, physiological range 85–90°) or the medial proximal tibial angle (MPTA, physiological range 85–90°), were documented pre- and postoperatively.

Percutaneous osteotomies were performed using a 4.0-mm drill and a chisel for completion, which causes minimal thermal damage to the bone and preserves the periosteal sleeve, for optimal callus formation.

Every patient received detailed oral and written instructions on the use of the device as well as an individual schedule with timing, rate, and total amount of lengthening. We aimed at starting distraction 5 days postoperatively at a distraction rate of 1.3 mm/day in femurs and starting 7 days postoperatively at 1.0 mm/day in tibias.

All patients were counseled regularly regarding the device and were followed radiographically on a biweekly basis during and at 4-week intervals after distraction until full consolidation. The range of motion of hip, knee, and ankle joints was recorded in addition to pain (visual analog scale), wound status, and complications. All patients underwent an intensive rehabilitation program under the guidance of physiotherapists not any less frequently than twice a week, at least until consolidation.

Consolidation was defined as corticalization of at least 3 sides of the callus on the biplane radiographs, with subsequent transition to full weight bearing. Insufficient callus formation was defined as a consolidation period more than 3 times longer than the distraction period (femur) or more than 4 times longer (tibia).

**Figure 2.**
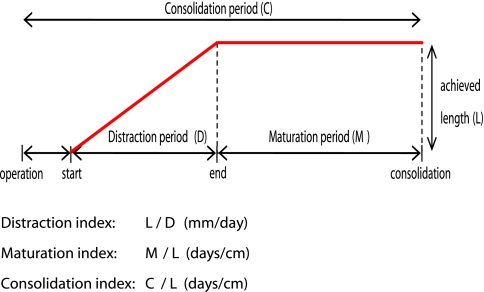
Overview of the different indices.

Every single lengthening period (distraction, maturation, consolidation) and the appropriate indices (Figure 2) were calculated and evaluated, with respect to the anatomical location, previous operations at the affected site, and age older or younger than 16.

Leg lengthening was considered successful in cases with no residual relevant discrepancy of ± 5 mm. For deformity correction in the frontal plane, the mechanical axis deviation (MAD) was evaluated preoperatively and intraoperatively with respect to the contralateral side and/or normal physiological conditions (Paley et al. 1994). Intraoperatively, mechanical axis alignment was determined by use of a grid plate with radio-opaque straight lines, which was placed underneath the patient on a radiolucent table. The Paley outcome score system designed for femora was used (Paley et al. 1997) and a modified version introduced by Krieg et al. (Krieg et al. 2008) was used for tibias.

Complications were differentiated as problems (grade 1), obstacles (grade 2), and minor or major complications (grade 3) (Paley 1990).

The calculation of total costs for a Fitbone treatment was based on the current local reimbursement rates for a public patient, 1 segment affected, implantation and explantation, hospital stay of 3 weeks in total (implantation and explantation), and physiotherapy twice a week for 1 year. Likewise, overall costs for leg length equalization by means of orthotic devices were calculated by a local orthopedic technician assuming a non-private patient with a leg length discrepancy of more than 2 cm requiring shoe lifts and necessary additional insoles twice a year for 60 years. In addition, implant costs for the Taylor Spatial Frame were taken from the manufacturer's price list.

The median follow-up time was 16 months (IQR: 12–22; range 12–27 months). By the end of the observation period, all but 2 nails had been explanted after a median of 14 months (IQR: 13–17; range 6.6–23 months).

### Statistics

Data are given as means, medians, and interquartile range (IQR). Comparisons between patient subgroups were assessed using Fisher's exact test for binary endpoints and by the Mann-Whitney U-test for continuous endpoints. A p-value of ≤ 0.05 was taken to indicate statistical significance. Statistical analysis was performed using SPSS software version 17.0.

## Results

### Length gain

Leg length equalization was successful in 30 of 32 cases (± 5mm). 2 showed a remaining LLD of 7 mm and 10 mm, which was clinically irrelevant. At the femur, the target length was achieved with a standard deviation of ± 6.1%. Regarding the tibia, the equalization was accomplished with a standard deviation of ± 7.3%.

### Distraction index and hospital stay

Distraction was started at an average of 6 (3–12) days postoperatively with a median distraction index of 1.0 (IQR: 0.9–1.2) mm/day and a median distraction period of 30 (IQR: 28–42) days. The median hospital stay was 12 (IQR: 10–14) days.

### Maturation

There was no statistically significant difference in the maturation index in terms of the influencing variables examined.

The median maturation period was 75 (IQR: 60–106) days for femurs (n = 21) and 96 days (IQR: 66–112) for tibias (n = 11). The median maturation index was 22 days/cm (IQR: 16–30) for femurs and 36 (IQR: 22–40) days/cm for tibias.

### Consolidation

The median consolidation index was 35 (IQR: 27–44) days/cm for the femur and 48 (IQR: 34–63) days/cm for the tibia. A statistically significant difference between femurs and tibias was seen, but not regarding other influencing variables.

### Deformity corrections

The median preoperative mechanical axis deviation (MAD) in patients with a varus axis (n = 8) was 13 (6–50) mm medially to the center of the knee joint, whereas the median postoperative MAD was 4 mm medially (38 mm medially to 11 mm laterally). In patients with a valgus axis (n = 9), the median preoperative MAD was 13 (5–54) mm laterally as compared to a postoperative median of 0 mm (10 mm medially to 28 mm laterally).

In patients with additional rotatory and/or sagittal corrective osteotomies (n = 5), a bilaterally equal range of motion of the hip and knee joint was seen immediately postoperatively (Table 1).

### Joint motion

**Figure 3.**
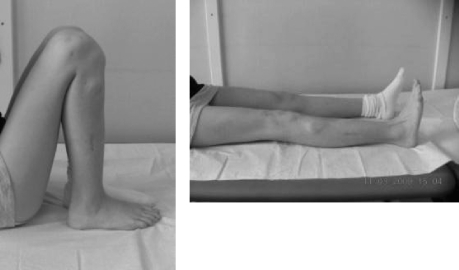
Range of motion 4 months postoperatively, after 5.5 cm of lengthening of the tibia.

The functional outcome was excellent in 26 patients, good in 5, and fair in 1. All but one had regained or even improved upon their preoperative range of motion of the hip, knee, and ankle joint (n = 8) (Figure 3).

### Complications

No intraoperative complications and no postoperative bone or soft tissue infections occurred. We observed 3 problems, 3 obstacles, and 4 complications (3 minor, 1 major) in 8 patients (Table 1). Insufficient callus formation was found in 2 tibias of smokers who both refused weight bearing. One (patient 14) was treated with autologous tricortical iliac bone grafting 8 months postoperatively. The other showed slow consolidation without need of further operative intervention.

In 1 case with a history of lengthening by external fixation (patient 12), a progressive knee extension-contracture required manual mobilization under anesthesia 5 months after the end of distraction.

One motor stopped because of overstraining of the mechanical implant by full weight bearing early on the patient's own authority, during the initial distraction period and after discharge from the hospital. It had to be exchanged, but this did not affect the final result. In 3 cases we observed running back of the telescopic part of the nail during the initial consolidation phase, which was twice associated with loosening of locking bolts. Another bolt loosened without loss of length. The persisting equinus of the foot in 1 patient (patient 24) was successfully corrected by a combined calcaneus and talus osteotomy using the Taylor Spatial frame.

### Costs

Total costs of approximately 100,000 euros were calculated to equalize a leg length discrepancy of more than 2 cm by means of orthotic devices. For a Fitbone treatment, total costs of approximately 38,000 euros were calculated (see Patients and methods). Pure implant costs amount to approximately 11,000 euros for the Fitbone nail, and to 7,500 euros for a standard Taylor Spatial Frame.

## Discussion

The use of mechanically driven intramedullary nails for limb lengthening and deformity correction has been reported repeatedly (Baumgart et al. 1997, Cole et al. 2001, Guichet 1999, Hankemeier et al. 2005, Guichet et al. 2003, Leidinger et al. 2006, Krieg et al. 2008, Betz et al. 1990, Garcia-Cimbrelo et al. 2002, Singh et al. 2006, Baumgart et al. 2006). The ISKD and the Albizzia are mechanically driven nails and provide all the advantages of intramedullary leg lengthening (Cole et al. 2001). Distraction with the ISKD is achieved by physiological gait movements. Thus, the distraction rate is less predictable, which may contribute to insufficient callus formation (Leidinger et al. 2006). In contrast, the Albizzia sometimes causes severe pain and discomfort at the osteotomy site since lengthening is accomplished by rotational maneuvers. Some authors have even reported re-admission for distraction under anesthesia (Garcia-Cimbrelo et al. 2002, Guichet et al. 2003). The Fitbone nail is the only intramedullary motorized lengthening device. In order to avoid high complication rates, the distribution was limited to a small number of selected surgeons by the company during the thorough implant development process.

Since most peer-reviewed literature on intramedullary lengthening involves a small number of patients (Table 2), we considered that it would be useful to add this relatively large case series.

**Table 2.** Summary of case series in peer-reviewed literature

Author	Device	No. of cases
Baumgart et al. 2005	motorized nail	12
Cole et al. 2001	mechanical nail	18
Hankemeier et al. 2005	mechanical nail	4
Guichet et al. 2003	mechanical nail	31
Leidinger et al. 2006	mechanical nail	22
Krieg et al. 2008	motorized nail	8
Betz et al. 1990	motorized nail	3
Garcia-Cimbrelo et al. 2002	mechanical nail	23
Singh et al. 2006	motorized nail	10

**Figure 4.**
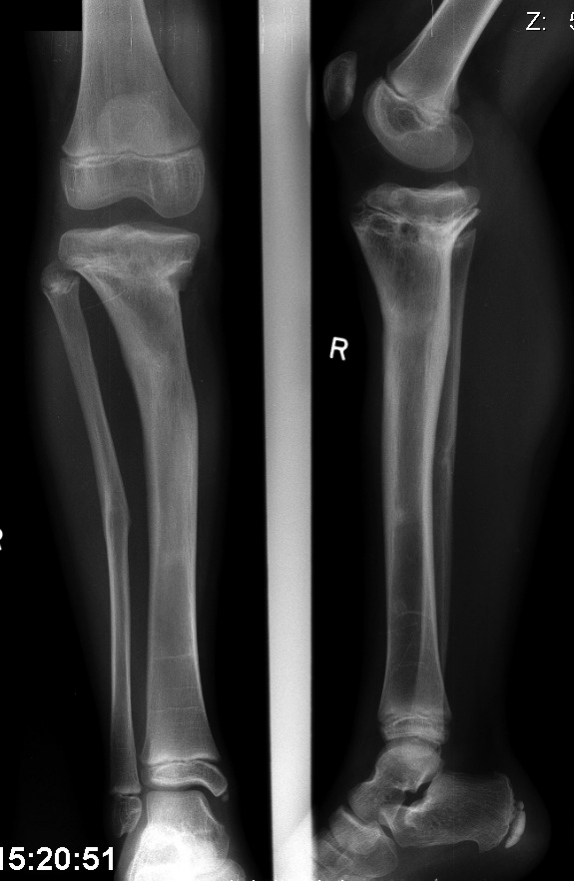
Frontal plane double deformity of the tibia with LLD of 5.5 cm in a patient with a history of osteomyelitis in early childhood and of previous operations.

**Figure 5.**
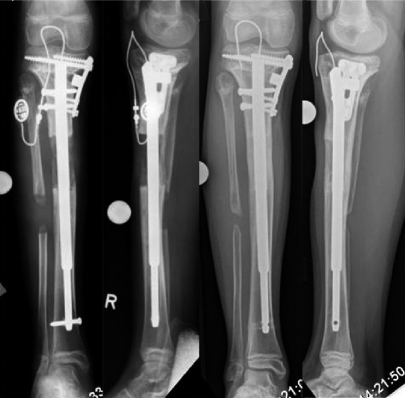
Anteroposterior and lateral radiographs 3 months (left) and 9 months (right) after surgery.

Unlike the application of external fixators, a solid intramedullary device requires defined anatomical preconditions that have to be adhered to, both during the preoperative planning and intraoperatively. However, for the accomplishment of corrections in 3 dimensions, reaming of the intramedullary cavity with straight and rigid reamers (not flexible ones) is as a prerequisite. Only the use of rigid reamers, which do not follow the line of minor resistance, allows to carry out the correlations anticipated during preoperative planning. In this context, adequate medullar dimensions respecting a minimal nail diameter of 11 mm at its shaft are one of the most important limitations of the Fitbone TAA. Further restrictions derive from its short overall length and a mandatory osteotomy level 7–11 cm from the joint line to ensure stable interlocking conditions. Marked angular deformities and a center of rotation and angulation far from the planned osteotomy are additional geometric obstacles, and may make segmental translation unavoidable (Figure 4). Axis alignment or implant position cannot be changed postoperatively. Hence, intraoperative acknowledgment of the preoperative planning is important (Baumgart (2009). In contrast, external fixators—and particularly the Taylor Spatial Frame (TSF)—are more forgiving by allowing postoperative and even computer-assisted adjustments (Blondel et al. 2009). However, even complex deformities may be overcome (Figure 5).

Application of solid lengthening nails in children is limited by open growth plates. However, data on children treated for tumors and also unpublished observations of experienced surgeons (regarding limb lengthening) suggest that the growth potential may remain unaffected when smooth polished implants are inserted through the central portion of a physis (Neel et al. 2004, Baumgart et al. 2006).

In keeping with the results of Fischgrund et al. (1994), we found a statistically significant difference in the consolidation index of patients with femoral lengthening and of those with tibial lengthening. However, as additional consideration of the maturation index did not reveal any statistically significant disparity, this may rather be explained by an earlier distraction start and higher daily rates at the femur. It is our experience that a distraction rate of about 1.3 mm/day at the femur is well accepted and sometimes even required in children, due to the enormous regeneration potential. This might be due to the excellent soft tissue coverage and the gentle osteotomy technique, which further contribute to rapid bone formation and consolidation.

Without transfixation of skin and muscles, lengthening nails facilitate full joint motion in the first few days after surgery, whereas external fixators decrease the range of motion even before the lengthening process has started (Maffulli et al. 2001). Thus, re-establishment of full range of motion with external fixators mostly exceeds rehabilitation times with intramedullary devices by far, as seen in our study.

The complication rates of external fixators range from 24% to 117% (Paley 1990, Tjernstrom et al. 1994, Paley et al. 1997, Blondel et al. 2009) and from 11% to 47% (Cole et al. 2001, Garcia-Cimbrelo et al. 2002, Guichet et al. 2003, Leidinger et al. 2006) for mechanically driven intramedullary devices, depending on the distraction length and the experience of the surgeon.

A complication rate of 12.5% in our series (4 of 32 patients) is in keeping with the findings of Baumgart et al. 2006) who reported a 13% complication rate in a larger number of patients (n = 150) treated with Fitbone TAA and SAA nails. These low complication rates may be attributed to the electronically-actuated lengthening mechanism and its reliable controllability—with consistent distraction rates as a consequence.

Technical complications or implant breakage rarely occur with Fitbone. In the series of 150 patients, Baumgart et al. (2005) reported technical difficulties in 6% of cases and implant breakage in 5%, with only a 3% rate of implant exchanges in total. Partial loss of gained length, as seen in 3 of our 32 patients, may occur due to loosened locking bolts or running back of the telescoping part. The latter will presumably be prevented now with the new generation of nails that have a running-back barrier.

The overall cost of lengthening with Fitbone was less than for conservative treatment, assuming the conditions mentioned earlier. Compared to external fixators such as the Taylor Spatial Frame, the Fitbone nail is somewhat more expensive. However, the much higher complication rate of external fixation—probably associated with additional hospital stays, medication (pain relief, antibiotics), and physiotherapy sessions—warrant differentiation between pure implant costs and costs of treatment, in addition to “soft” factors such as cosmetics, patient satisfaction, and quality of life during and after the lengthening procedure (Tjernstrom et al. 1994, Paley 1990, Song et al. 2005).

Paley et al. (1997), who published a similar cost report, put forward mean costs of about $24,000 ($18,000 to $28,000) for unilateral femoral lengthening over an intramedullary nail compared to $19,000 ($18,000 to $29,000) for unilateral Ilizarov femoral lengthening. Likewise, they included hospital charges as well as fees for the index procedure and removal of the external fixator (factors such as duration of hospital stay were not listed in detail) but did not include the costs for rehabilitation and additional costs due to complications. Even so, these considerations should be taken into account in order to compare overall costs. Healthcare providers especially—who tend to refuse reimbursement of new and at first sight expensive innovative methods—should be made to understand that such treatment options may prove to be less expensive if the wider picture of overall cost is taken into account.

Apart from the explicit desire of the majority of our patients to have the implants removed after the lengthening procedure, we consider the removal mandatory in order to avoid difficulties in the future, such as removal of an ingrown nail or interference with arthroplastic devices later on. Furthermore, the analysis of each explanted device by the manufacturer contributes to the quality of management and promotes development and perfection of the nail.

Our study was limited by the lack of a matched control group, treated with different lengthening devices. Furthermore, long-term results on lengthening with Fitbone nails are still not available.

In summary, the outcome achieved in our patients is a respectable result, and there was a low complication rate. There was a significant difference in the consolidation index in the treatment of femurs and tibias. The high implant costs are compensated for by competitive treatment costs. Intramedullary leg lengthening is, however, a demanding procedure.
